# Supplementary material for: Predicting visual function by interpreting a neuronal wiring diagram
Source: Nature. 2024 Oct 2;634(8032):113–23. doi: 10.1038/s41586-024-07953-5 (PMC11446822; doi:10.1038/s41586-024-07953-5)
Supplement: Supplementary file 6 — CRF and ERF predictions for individual TmY4 and TmY9 cells. Analogous to Supplementary Data 3, but for TmY target types. Shown are the top four monosynaptic pathways, the strongest pathway passing through each of the top ten intermediary types (ranking from Extended Data Fig. 7), and the trisynaptic pathway Tm1–TmY–Dm3–TmY (see the section entitled Prediction of spatial normalization). [file 41586_2024_7953_MOESM6_ESM.zip › DataS4/TmY9qΓèÑ/720575940629196818.pdf]

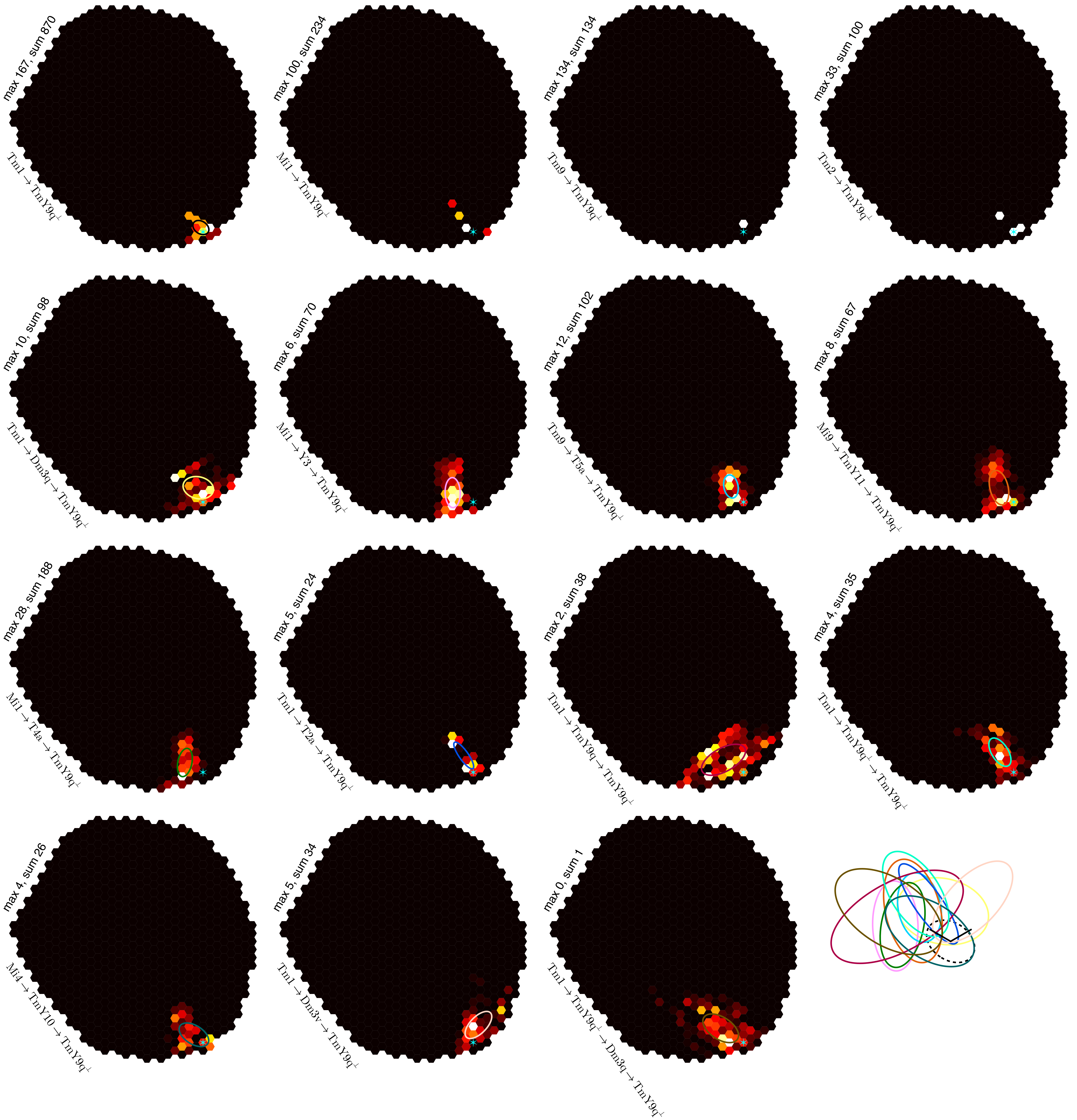
max 167, sum 870
Tm1 → TmY9q⊥
max 100, sum 234
Mi1 → TmY9q⊥
max 134, sum 134
Tm9 → TmY9q⊥
max 33, sum 100
Tm2 → TmY9q⊥
max 10, sum 98
Tm1 → Dm3q → TmY9q⊥
max 6, sum 70
Mi1 → Y3 → TmY9q⊥
max 12, sum 102
Tm9 → T5a → TmY9q⊥
max 8, sum 67
Mi9 → TmY11 → TmY9q⊥
max 28, sum 188
Mi1 → T4a → TmY9q⊥
max 5, sum 24
Tm1 → T2a → TmY9q⊥
max 2, sum 38
Tm1 → TmY9q → TmY9q⊥
max 4, sum 35
Tm1 → TmY9q⊥ → TmY9q⊥
max 4, sum 26
Mi4 → TmY10 → TmY9q⊥
max 5, sum 34
Tm1 → Dm3v → TmY9q⊥
max 0, sum 1
Tm1 → TmY9q⊥ → Dm3q → TmY9q⊥
